# Evaluating Variation in Lymph Node Sampling During Sentinel Lymph Node Biopsy for Melanoma

**DOI:** 10.1245/s10434-025-18063-5

**Published:** 2025-08-16

**Authors:** Crystal D. Taylor, Vanessa S. Niba, Alison S. Baskin, Nicole M. Mott, Erin Kim, Abigail Kappelman, Ton Wang, Brandy R. Sinco, Amanda B. Francescatti, Daniel J. Boffa, Samantha Hendren, Judy C. Boughey, Tina J. Hieken, Ronald J. Weigel, Tasha M. Hughes, Lesly A. Dossett

**Affiliations:** 1 Department of Surgery, Michigan Medicine, Ann Arbor, MI USA; 2 Center for Health Outcomes and Policy, Michigan Medicine, Ann Arbor, MI USA; 3 Institute for Healthcare Policy and Innovation, University of Michigan, Ann Arbor, MI USA; 4 National Clinician Scholars Program, University of Michigan, Ann Arbor, MI USA; 5 Department of Surgery, University of California San Francisco, San Francisco, CA USA; 6 Department of Surgery, University of Colorado, Aurora, CO USA; 7 Veterans Affairs Center for Clinical Management Research, Ann Arbor, MI USA; 8 University of Michigan Medical School, Ann Arbor, MI USA; 9 Department of Surgery, Duke University, Durham, NC USA; 10 American College of Surgeons Cancer Research Program, Chicago, IL USA; 11 Department of Surgery, Section of Thoracic Surgery, Yale University School of Medicine, New Haven, CT USA; 12 Department of Surgery, Indiana University, Indianapolis, IN USA; 13 Division of Breast and Melanoma Surgical Oncology, Department of Surgery, Mayo Clinic, Rochester, MN USA; 14 Department of Surgery, University of Iowa, Iowa City, IA USA; 15 Division of Surgical Oncology, University of Michigan, Ann Arbor, MI USA

## Abstract

**Background:**

The operative standard for melanoma, implemented by the Commission on Cancer (CoC), addresses margin width and excision depth, but does not collect information on sentinel lymph node biopsy (SLNB). However, SLNB, an implemented technical standard in breast cancer, is also critical in the management of melanoma through its impact on nodal staging. This study aimed to characterize the current facility-level variation in nodal yield and nodal positivity to determine if there is an opportunity for improvement through standardization.

**Patients and Methods:**

Using the National Cancer Database, we identified patients with T1b–T4 melanoma of the trunk and upper extremities who underwent SLNB from 2018 to 2022. Reliability-adjusted estimates for nodal yield and nodal positivity were calculated using Poisson regression and logistic regression with random intercepts for hospitals.

**Results:**

We identified 48,653 melanoma patients from 1167 facilities. SLNB median nodal yield was 2.4 (IQR 2.2–2.7), ranging from 1.4 to 7.0. SLNB median nodal positivity was 18.0% (IQR 17.1–19.5%), ranging from 11.6 to 40.5%. A weak correlation between nodal yield and nodal positivity was observed (Spearman correlation coefficient = 0.08, *p* = 0.009).

**Conclusions:**

Facility-level variation in nodal yield was minimal and weakly correlated with nodal positivity. This suggests that SLNB performed for melanoma of the trunk and upper extremities is well standardized across CoC hospitals in the absence of a defined operative standard. Future efforts to improve the quality of melanoma nodal surgery may be best focused on technical elements of other procedures, such as lymphadenectomy or more novel lymph node dissection approaches following neoadjuvant therapy.

**Supplementary Information:**

The online version contains supplementary material available at 10.1245/s10434-025-18063-5.

The *Operative Standards for Cancer Surgery*, published by the American College of Surgeons Cancer Research Program, provide a unique opportunity to enhance quality in cancer care by improving surgical technical quality.^1,2^ These series of manuals contain more than 150 critical elements of commonly performed cancer operations that should be executed by practicing surgeons. The Commission on Cancer (CoC) incorporated six of these operative standards into its accreditation standards for facilities (Standards 5.3–5.8).^3^ As compliance with these implemented standards is expected by CoC-accredited facilities and additional standards will likely be added, it is critical to understand areas of technical variation that may be potential targets for improvement. For cutaneous melanoma, the operative standard (Standard 5.5) addresses wide local excision.^4^ This standard is based on evidence that adequate margins and depth during excision decreased local recurrence, and performing an excision with the smallest necessary margin was associated with decreased wound morbidity and improved patient quality of life.^5–10^ In contrast to other implemented standards, which address a component of lymph node evaluation, no other implemented standard currently exists for melanoma.

Nodal staging, performed through sentinel lymph node biopsy (SLNB), is important in melanoma as it directly affects overall staging, prognostication, and adjuvant therapy decisions. While nodal staging has not been incorporated as an operative standard for melanoma, the CoC Operative Standards for breast cancer address SLNB and axillary lymph node dissection (Standards 5.3 and 5.4).^4^ In early-stage breast cancer, adjuvant therapy decisions are becoming less reliant on nodal staging as gene expression profiling is increasingly used to predict prognosis and benefit from adjuvant chemotherapy.^11^ However, in melanoma, no corollary currently exists, and thus, accurate nodal staging using SLNB is critical as nodal positivity is the main indication for adjuvant therapy treatments. This suggests a need to understand the current level of variation in SLNB yield and its correlation to nodal positivity in melanoma as a target for quality improvement, which is not yet known.

As the value of nodal evaluation has been recognized for other cancer types within the operative standards, determining whether SLNB staging in melanoma is standardized throughout CoC-accredited facilities represents a critical area for investigation. The objective of our study was to characterize the current facility-level variation in lymph node yield and nodal positivity during SLNB in melanoma. Understanding any potential variation in melanoma can help ascertain if there is potential room for quality improvement, and thus, whether standardization would be valuable. Alternatively, if this procedure is well standardized for melanoma, then quality improvement efforts could focus on other technical elements within melanoma surgery.

## Patients and Methods

We performed a retrospective cohort study using the National Cancer Database (NCDB) to identify patients 18 years and older undergoing SLNB for melanoma from 2018 to 2022. The University of Michigan Institutional Review Board deemed this study exempt owing to the use of deidentified information, and patient informed consent was not obtained. This report follows the Strengthening the Reporting of Observational Studies (STROBE) guidelines for cohort studies.^12^

### Data Source

The NCDB is a national clinical oncology database containing hospital registry data collected from CoC-accredited facilities.^13^ It is the largest clinical cancer registry in the world, containing over 34 million records, and captures approximately 74% of new cancer diagnoses.^14–16^

### Study Population

The cohort included men and women aged 18 years and older who underwent SLNB for T1b–T4 cutaneous melanoma of the trunk and upper extremities from 2018 to 2022. Melanoma of the trunk and upper extremities was chosen to limit the variation observed by restricting SLNB to the axilla only. This created a similar cohort as previously published for SLNB in breast cancer, which is also performed in the axilla.^17^ Patients with clinically node-positive disease, metastatic disease, or those receiving treatment outside of their primary facility were excluded. Missing observations based on our selection criteria were omitted from this study. Hospital volume was based on the average annual melanoma case count and categorized as low (1–8 cases), medium (8–50 cases), or high (≥ 51 cases).

### Exposure

Patients undergoing SLNB for melanoma, with at least one lymph node sampled, were identified using the *Sentinel Lymph Nodes Examined* variable in the NCDB, which was unavailable before 2018. This variable was also used to quantify the number of lymph nodes removed during SLNB. While the NCDB includes lymph node values up to 90 or more, we truncated possible lymph nodes examined to 40 (over 40 were considered inaccurate and excluded). The *Sentinel Lymph Nodes Positive* variable was used to quantify the number of positive nodes obtained following SLNB.

### Outcome Variables

Our primary outcomes were the mean, median, and range of lymph nodes examined, and the nodal positivity rate at each facility for patients undergoing SLNB for melanoma. Our secondary outcome was to evaluate the correlation between the number of lymph nodes examined and the nodal positivity rate for SLNB by facility.

### Statistical Analysis

Reliability-adjusted estimates for count variables, such as lymph node yields, were generated from Poisson regression models, with random intercepts for each hospital, also known as generalized linear mixed models (GLMM). Similarly, the reliability-adjusted estimates for binary outcomes, such as one or more positive lymph nodes, were computed from GLMM for logistic regression with random intercepts for hospitals. A GLMM with a hospital random intercept accounts for clustering within hospitals and is equivalent to an empirical Bayesian estimate.^18^

Key reliability-adjusted estimates for mean lymph node yields and nodal positivity are displayed on caterpillar plots (Figs. 2 and 3). Reliability-adjusted estimates for mean lymph nodes examined and nodal positivity are displayed on a scatter plot (Fig. 4). The Spearman correlation coefficient was used to calculate the correlation between the mean lymph nodes examined and nodal positivity because Spearman correlation detects any increasing or decreasing trends, even if the trend is not linear.^19^

Finally, to evaluate which facility-level characteristics were associated with nodal yield and nodal positivity, analysis of variance (ANOVA) was performed on the facility-level estimates by volume category, facility type, and location. A *p*-value of less than 0.05 was considered significant, and all *p*-values were two-sided. All analyses were performed using SAS statistical software version 9.4 (SAS Institute Inc., Cary, NC).

## Results

### Study Population

**Fig. 1:**
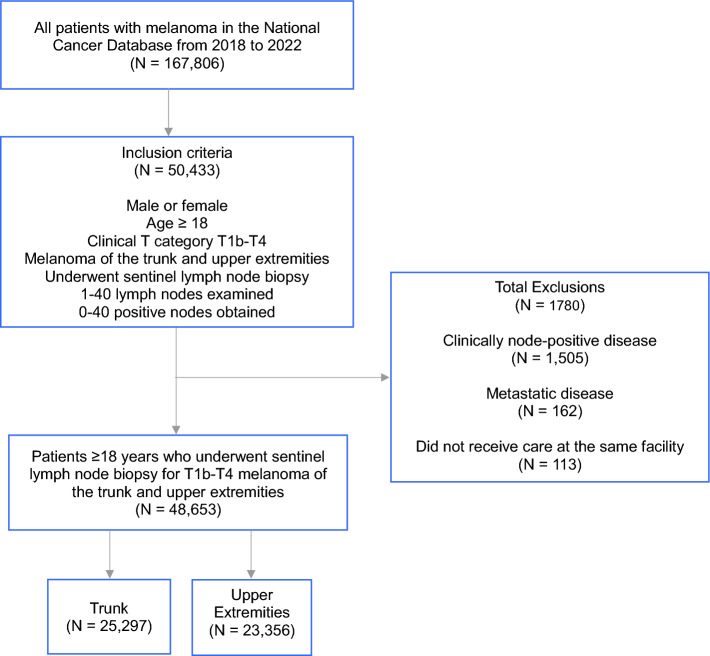
Study consort diagram

A total of 48,653 men and women undergoing SLNB for melanoma of the trunk and upper extremities across 1167 facilities were included (Fig. 1). The median age of the cohort was 65 years (interquartile range [IQR] 54–73). Overall, 52% (*n* = 25,297) of patients had melanoma of the trunk while 48% (*n* = 23,356) had melanoma of the upper extremities. Patient distribution by facility characteristics is noted in Table 1. In sum, 41% (*n* = 20,046) of patients were treated at an academic center, 28% (*n* = 13,406) at a comprehensive cancer center, 20% (*n* = 9619) at an integrated network, and 4% (*n* = 1925) at a community cancer center. Most patients were treated at high volume facilities (60%, *n* = 29,317).

**Table 1:** Characteristics of patients with melanoma of the trunk and upper extremities undergoing sentinel lymph node biopsy

Characteristic	*N* (%) or mean
Age, years, mean (SD)	63.0 (14.2)
Age, years, median (IQR)	65 (54, 73)
Sex	
Female	18,794 (38.6%)
Male	29,859 (61.4%)
Race	
White	47,737 (98.1%)
Black	124 (0.3%)
Asian	103 (0.2%)
Native American	95 (0.2%)
Other Race or Unknown	594 (1.2%)
Hispanic Ethnicity	587 (1.2%)
Not Hispanic/LatinX	47,364 (97.4%)
Unknown	702 (1.4%)
Tumor characteristics	
Clinical T	
T1b	11,274 (23.2%)
T2	18,491 (38.0%)
T3	10,327 (21.2%)
T4	5566 (11.4%)
Unknown	2995 (6.2%)
Clinical N category	
N0	45,593 (93.7%)
Unknown	3060 (6.3%)
Tumor location	
Trunk	25,297 (52.0%)
Upper extremity	23,356 (48.0%)
Facility type	
Community cancer	1925 (4.0%)
Comprehensive center	13,406 (27.6%)
Academic center	20,046 (41.2%)
Integrated network	9619 (19.8%)
Unknown	3657 (7.5%)
Annual case volume	
Low (< 8)	3022 (6.2%)
Medium (8–50)	16,314 (33.5%)
High (≥ 51)	29,317 (60.3%)

*SD* standard deviation, *IQR* interquartile range

### Lymph Nodes Examined

**Fig. 2:**
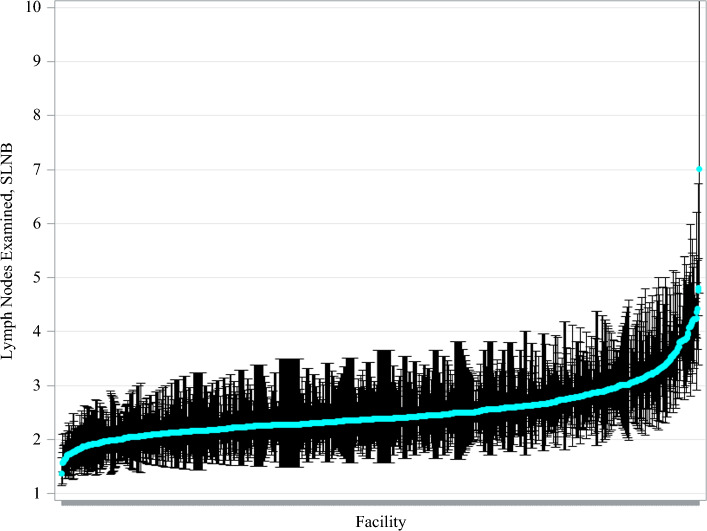
Facility-level variation in nodal yield during sentinel lymph node biopsy for melanoma of the trunk and upper extremities from 2018 to 2022

Facility-level lymph node yield is summarized in Table 2. The median number of lymph nodes removed during SLNB for melanoma of the trunk and upper extremities was 2.4 (IQR 2.2–2.7) and ranged from 1.4 to 7.0 across facilities. Facility-level variation in lymph node yield for SLNB in melanoma is displayed in Fig. 2. High volume facilities had a slightly higher mean lymph node yield than medium and low volume facilities (2.6 for high volume versus 2.5 for medium and low volume, *p* = 0.037; Supplementary Fig. 1 and Table 1). There were no statistically significant differences in mean lymph node yield between facility type.

**Table 2:** Facility-level variation in nodal yield and nodal positivity for melanoma of the trunk and upper extremities during sentinel lymph node biopsy

Mean (SD)	Median (IQR)	Range
*Lymph node yield (estimates)*		
2.5 (0.5)	2.4 (2.2–2.7)	1.4–7.0
*Nodal positivity rate (percentages)*		
18.4 (2.6)	18.0 (17.1–19.5)	11.6–40.5

### Nodal Positivity Rate and Correlation between Nodal Yield and Nodal Positivity

**Fig. 3:**
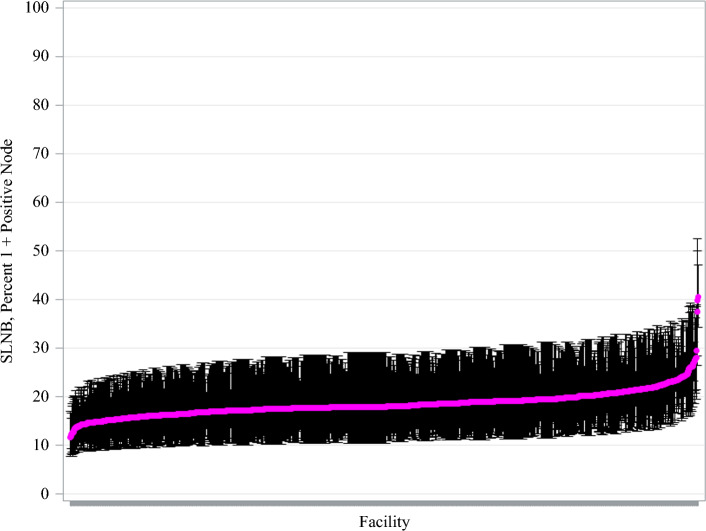
Facility-level variation in mean lymph node positivity rate for melanoma of the trunk and upper extremities from 2018 to 2022

**Fig. 4:**
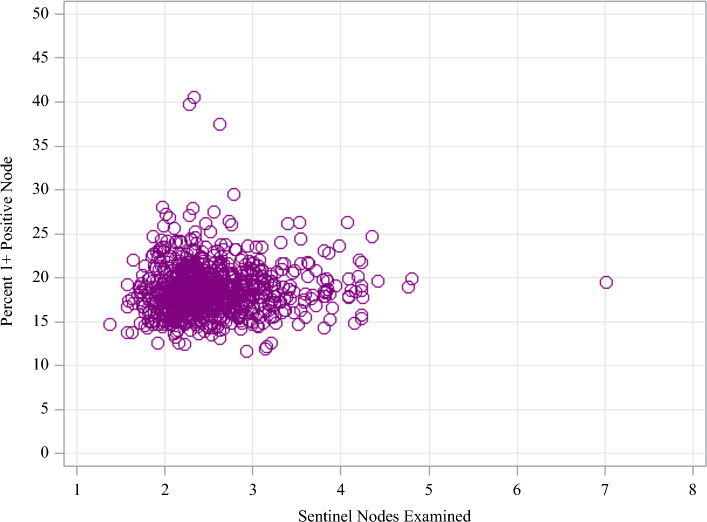
Scatterplot of reliability-adjusted lymph nodes examined by nodal positivity during sentinel lymph node biopsy for melanoma: Spearman correlation coefficient = 0.08

The median nodal positivity rate was 18.0% (IQR 17.1–19.5%) by facility. There was over a three-fold variation in the nodal positivity rate across facilities, with rates ranging from 11.6 to 40.5%. Facility-level variation in nodal positivity rates for SLNB in melanoma is displayed in Fig. 3. No statistically significant differences were observed in the nodal positivity rate based on facility volume. Nodal positivity rates were slightly higher for patients treated at academic centers compared with other facility types (positivity rate = 19%, *p* = 0.014; Supplementary Fig. 2 and Table 2). The correlation between the mean number of lymph nodes examined by facility and the rate of nodal positivity for SLNB in melanoma was weakly correlated (0.08, *p* = 0.009) (Fig. 4).

## Discussion

This study examined facility-level variation in lymph node yield and nodal positivity during SLNB for melanoma. These results aid in understanding the potential impact of the CoC Operative Standards by understanding the current variation in this procedure as a possible target for improving the quality of surgical management for melanoma. We observed some facility-level variation in lymph node yield for SLNB performed for melanoma of the trunk and upper extremities. Ultimately, this variation was minimal and weakly correlated with nodal positivity, suggesting quality improvement efforts towards this procedure as a technical standard would have minimal clinical impact on cancer quality.

SLNB has been an integral component of treatment for melanoma for over 30 years, providing critical information to guide adjuvant therapy decisions and locoregional control of disease.^20–24^ Known factors that contribute to variation in lymph node yield during SLNB for melanoma include age, sex, primary site of disease, histologic subtype, and Breslow thickness.^25^ Despite these known causes of variability, our findings support that SLNB performed for melanoma of the trunk and upper extremities is well standardized with minimal variation seen at the facility level. Similar levels of standardization have been observed in SLNB performed for breast cancer pre-implementation of CoC Operative Standard 5.3.^17^ This suggests that axillary SLNB is performed with minimal variability across malignancy types.

The overall nodal positivity rate observed in this study is consistent with national trends reported in prior studies.^20,21,26^ Nodal positivity in melanoma is known to be influenced by factors such as surgeon experience, pathologic examination, Breslow depth, lymphovascular invasion, age, presence of ulceration, and tumor site.^27,28^ Differences in nodal positivity rates were observed by facility type, with academic centers obtaining higher nodal positivity rates compared with other facilities. Interestingly, most patients in our study, as in breast cancer, were also treated at high-volume academic centers and comprehensive cancer centers, with only a small portion of patients treated at community cancer centers. This may reflect referral patterns for treatment by primary care physicians and dermatologists. Ultimately, this suggests that melanoma surgery is likely performed by surgeons with highly specialized training, irrespective of subspecialty. This specialized training may also extend to pathologists. Pathologic evaluation of lymph nodes for axillary SLNB in melanoma are subject to specific considerations to accurately identify disease, including appropriate sectioning of samples and using immunohistochemical staining to identify metastatic deposits.^23,29–32^ The concentration of melanoma surgery at these centers reflects the expertise within multidisciplinary care needed in the perioperative setting and may explain a relative lack of variation in some surrogates of technical quality.

Adjuvant therapies in melanoma, such as checkpoint inhibitors and targeted therapies with BRAF and MEK inhibitors, have substantially contributed to improvements in survival.^30,33–35^ Indications for adjuvant therapy include stage III and IV disease, intermediate thickness melanoma with ulceration, and thick melanoma with or without ulceration.^36,37^ It is important to note that patients with thin melanoma upstaged to limited node-positive disease (stage IIIa) paradoxically have a better prognosis compared with patients with stage IIb/c disease and do not universally receive adjuvant treatment.^38,39^ Nonetheless, lymph node evaluation provides useful information to guide treatment conversations with patients, and appropriate upstaging may provide a survival benefit even if no additional treatments are administered.

The implementation of the Operative Standards by the CoC into its accreditation process represents an important effort to improve the quality of cancer surgery. Understanding key potential targets for improvement is critical to the mission of the program. We have demonstrated that SLNB technical performance for melanoma, as previously seen in breast cancer, is well standardized, likely reflecting that a ceiling effect has been reached for this procedure. As such, efforts to target SLNB for melanoma as a CoC Operative Standard would not likely improve surgical quality, which is the primary goal of the program. Future efforts to improve the technical quality of melanoma nodal surgery may consider focusing on other technical elements, such as lymphadenectomy or targeted lymph node dissections following neoadjuvant therapy.

Important limitations should be noted as they relate to our study. The NCDB reports data from CoC-accredited hospitals, which only represent approximately 30% of all hospitals within the USA and could limit the generalizability of our results for patients treated for melanoma at non-CoC-accredited facilities. However, as the NCDB encompasses 74% of new cancer diagnoses in the USA and is the largest cancer registry in the world, it likely accurately reflects the quality for most patients receiving surgery for melanoma. The results of this study may not be applicable to SLNB performed for sites other than the axilla, as factors such as drainage to multiple lymph node basins are known to affect variation in lymph node yield.^40–43^ Consequently, these findings may not be representative of the current facility-level variation for this procedure in melanoma overall. However, we purposefully selected for a cohort of axillary SLNB in melanoma so that it would be similar to previously reported data on a similar cohort in breast cancer. Evaluating SLNB at a specific anatomic site allows for a more accurate evaluation of performance among facilities by removing potential differences due to anatomic variation.

## Conclusions

Minimal facility-level variation was present in lymph node yield for SLNB performed in the axilla. This variation weakly correlated with nodal positivity, suggesting that SLNB performed for melanoma of the trunk and upper extremities is already well standardized across CoC hospitals in the absence of a defined standard. Future efforts to improve the quality of melanoma nodal surgery would be better focused on technical elements such as lymphadenectomy or targeted procedures following neoadjuvant therapy.

## Supplementary Information

Below is the link to the electronic supplementary material.

Supplementary file 1 (DOCX 75 kb)

## References

[1] Nelson H, Hunt KK, Alliance for Clinical Trials in Oncology. Operative standards for cancer surgery: volume 1. Philadelphia: Lippincott Williams & Wilkins; 2015.

[2] Katz MH, American College of Surgeons Clinical Research Program. Operative standards for cancer surgery: volume 2. Philadelphia: Lippincott Williams & Wilkins; 2018.

[3] Katz MHG, Francescatti AB, Hunt KK, Cancer Surgery Standards Program of the American College of Surgeons. Technical standards for cancer surgery: commission on cancer standards 5.3–5.8. *Ann Surg Oncol*. 2022;29(11):6549–58. 10.1245/s10434-022-11375-w. 35187620 10.1245/s10434-022-11375-w

[4] American College of Surgeons Commission on Cancer. Optimal resources for cancer care (2020 standards). *American College of Surgeons Commission on Cancer: Chicago, IL, USA*. 2020.

[5] Balch CM, Urist MM, Karakousis CP, et al. Efficacy of 2-cm surgical margins for intermediate-thickness melanomas (1 to 4 mm). Results of a multi-institutional randomized surgical trial. *Ann Surg*. 1993;218(3):262–7. 10.1097/00000658-199309000-00005. (**discussion 267-9**). 8373269 10.1097/00000658-199309000-00005 PMC1242959

[6] Gillgren P, Drzewiecki KT, Niin M, et al. 2-cm versus 4-cm surgical excision margins for primary cutaneous melanoma thicker than 2 mm: a randomised, multicentre trial. *Lancet*. 2011;378(9803):1635–42. 10.1016/S0140-6736(11)61546-8. 22027547 10.1016/S0140-6736(11)61546-8

[7] Khayat D, Rixe O, Martin G, et al. Surgical margins in cutaneous melanoma (2 cm versus 5 cm for lesions measuring less than 2.1-mm thick). *Cancer*. 2003;97(8):1941–6. 10.1002/cncr.11272. 12673721 10.1002/cncr.11272

[8] Ringborg U, Andersson R, Eldh J, et al. Resection margins of 2 versus 5 cm for cutaneous malignant melanoma with a tumor thickness of 0.8 to 2.0 mm: randomized study by the Swedish Melanoma Study Group. *Cancer*. 1996;77(9):1809–14. 10.1002/(SICI)1097-0142(19960501)77:9%3c1809::AID-CNCR8%3e3.0.CO;2-6. 8646678 10.1002/(SICI)1097-0142(19960501)77:9<1809::AID-CNCR8>3.0.CO;2-6

[9] Thomas JM, Newton-Bishop J, A’Hern R, et al. Excision margins in high-risk malignant melanoma. *N Engl J Med*. 2004;350(8):757–66. 10.1056/NEJMoa030681. 14973217 10.1056/NEJMoa030681

[10] Veronesi U, Cascinelli N. Narrow excision (1-cm margin). A safe procedure for thin cutaneous melanoma. *Arch Surg*. 1991;126(4):438–41. 10.1001/archsurg.1991.01410280036004. 2009058 10.1001/archsurg.1991.01410280036004

[11] Bao T, Davidson NE. Gene expression profiling of breast cancer. *Adv Surg*. 2008;42:249–60. 10.1016/j.yasu.2008.03.002. 18953822 10.1016/j.yasu.2008.03.002 PMC2775529

[12] von Elm E, Altman DG, Egger M, et al. Strengthening the Reporting of Observational Studies in Epidemiology (STROBE) statement: guidelines for reporting observational studies. *BMJ*. 2007;335(7624):806–8. 10.1136/bmj.39335.541782.AD. 17947786 10.1136/bmj.39335.541782.AD PMC2034723

[13] Boffa DJ, Rosen JE, Mallin K, et al. Using the National Cancer Database for outcomes research: a review. *JAMA Oncol*. 2017;3(12):1722–8. 10.1001/jamaoncol.2016.6905. 28241198 10.1001/jamaoncol.2016.6905

[14] Bilimoria KY, Bentrem DJ, Stewart AK, Winchester DP, Ko CY. Comparison of commission on cancer-approved and -nonapproved hospitals in the United States: implications for studies that use the National Cancer Data Base. *J Clin Oncol*. 2009;27(25):4177–81. 10.1200/JCO.2008.21.7018. 19636004 10.1200/JCO.2008.21.7018

[15] Bilimoria KY, Stewart AK, Winchester DP, Ko CY. The National Cancer Data Base: a powerful initiative to improve cancer care in the United States. *Ann Surg Oncol*. 2008;15(3):683–90. 10.1245/s10434-007-9747-3. 18183467 10.1245/s10434-007-9747-3 PMC2234447

[16] Palis BE, Janczewski LM, Browner AE, et al. The National Cancer Database conforms to the standardized framework for registry and data quality. *Ann Surg Oncol*. 2024;31(9):5546–59. 10.1245/s10434-024-15393-8. 38717542 10.1245/s10434-024-15393-8 PMC11300494

[17] Taylor CD, Wang T, Baskin AS, et al. American College of Surgeons Operative Standards and breast cancer outcomes. *JAMA Netw Open*. 2024;7(11):e2446345. 10.1001/jamanetworkopen.2024.46345. 39565622 10.1001/jamanetworkopen.2024.46345 PMC11579798

[18] Dimick JB, Ghaferi AA, Osborne NH, Ko CY, Hall BL. Reliability adjustment for reporting hospital outcomes with surgery. *Ann Surg*. 2012;255(4):703–7. 10.1097/SLA.0b013e31824b46ff. 22388108 10.1097/SLA.0b013e31824b46ff

[19] Spearman C. The proof and measurement of association between two things. By C. Spearman, 1904. *Am J Psychol*. 1987;100(3–4):441–71. 3322052

[20] Moncrieff MD, Lo SN, Scolyer RA, et al. Clinical outcomes and risk stratification of early-stage melanoma micrometastases from an international multicenter study: implications for the management of American Joint Committee on Cancer IIIA Disease. *J Clin Oncol*. 2022;40(34):3940–51. 10.1200/JCO.21.02488. 35849790 10.1200/JCO.21.02488

[21] Morton DL, Thompson JF, Cochran AJ, et al. Final trial report of sentinel-node biopsy versus nodal observation in melanoma. *N Engl J Med*. 2014;370(7):599–609. 10.1056/NEJMoa1310460. 24521106 10.1056/NEJMoa1310460 PMC4058881

[22] Morton DL, Thompson JF, Essner R, et al. Validation of the accuracy of intraoperative lymphatic mapping and sentinel lymphadenectomy for early-stage melanoma: a multicenter trial. Multicenter Selective Lymphadenectomy Trial Group. *Ann Surg*. 1999;230(4):453–63. 10.1097/00000658-199910000-00001. (**discussion 463-5**). 10522715 10.1097/00000658-199910000-00001 PMC1420894

[23] Morton DL, Wen DR, Wong JH, et al. Technical details of intraoperative lymphatic mapping for early stage melanoma. *Arch Surg*. 1992;127(4):392–9. 10.1001/archsurg.1992.01420040034005. 1558490 10.1001/archsurg.1992.01420040034005

[24] Crystal JS, Thompson JF, Multicenter Selective Lymphadenectomy Trials Study G, et al. Therapeutic value of sentinel lymph node biopsy in patients with melanoma: a randomized clinical trial. *JAMA Surg*. 2022;157(9):835–42. 10.1001/jamasurg.2022.2055. 35921122 10.1001/jamasurg.2022.2055 PMC9475390

[25] Lizalek JM, Dougherty CE, Santamaria-Barria JA, Reames BN, Foster J, Mammen JM. Impact of clinicopathologic factors on the number of lymph nodes examined in patients with melanoma. *Surg Oncol Insight*. 2025;2(1):100123.

[26] Miller JR III, Lo SN, Nosrati M, et al. Improving selection for sentinel lymph node biopsy among patients with melanoma. *JAMA Netw Open*. 2023;6(4):e236356. 10.1001/jamanetworkopen.2023.6356. 37074717 10.1001/jamanetworkopen.2023.6356 PMC10116363

[27] Jeremic J, Radenovic K, Jurisic M, et al. Primary melanoma histopathologic predictors of sentinel lymph node positivity: a proposed scoring system for risk assessment and patient selection in a clinical setting. *Medicina (Kaunas)*. 2023. 10.3390/medicina59111921. 38003969 10.3390/medicina59111921 PMC10673032

[28] Kinnier CV, Paruch JL, Dahlke AR, et al. Adjusted hospital sentinel lymph node positivity rates in melanoma: a novel potential measure of quality. *Ann Surg*. 2016;263(2):392–8. 10.1097/SLA.0000000000001052. 26488806 10.1097/SLA.0000000000001052

[29] Gershenwald JE, Colome MI, Lee JE, et al. Patterns of recurrence following a negative sentinel lymph node biopsy in 243 patients with stage I or II melanoma. *J Clin Oncol*. 1998;16(6):2253–60. 10.1200/JCO.1998.16.6.2253. 9626228 10.1200/JCO.1998.16.6.2253

[30] Roberts AA, Cochran AJ. Pathologic analysis of sentinel lymph nodes in melanoma patients: current and future trends. *J Surg Oncol*. 2004;85(3):152–61. 10.1002/jso.20028. 14991887 10.1002/jso.20028

[31] Spanknebel K, Coit DG, Bieligk SC, Gonen M, Rosai J, Klimstra DS. Characterization of micrometastatic disease in melanoma sentinel lymph nodes by enhanced pathology: recommendations for standardizing pathologic analysis. *Am J Surg Pathol*. 2005;29(3):305–17. 10.1097/01.pas.0000152134.36030.b7. 15725798 10.1097/01.pas.0000152134.36030.b7

[32] Veenstra HJ, Wouters MW, Kroon BB, Olmos RA, Nieweg OE. Less false-negative sentinel node procedures in melanoma patients with experience and proper collaboration. *J Surg Oncol*. 2011;104(5):454–7. 10.1002/jso.21967. 21538361 10.1002/jso.21967

[33] Long GV, Hauschild A, Santinami M, et al. Adjuvant dabrafenib plus trametinib in stage iii braf-mutated melanoma. *N Engl J Med*. 2017;377(19):1813–23. 10.1056/NEJMoa1708539. 28891408 10.1056/NEJMoa1708539

[34] Maio M, Lewis K, Demidov L, et al. Adjuvant vemurafenib in resected, BRAF(V600) mutation-positive melanoma (BRIM8): a randomised, double-blind, placebo-controlled, multicentre, phase 3 trial. *Lancet Oncol*. 2018;19(4):510–20. 10.1016/S1470-2045(18)30106-2. 29477665 10.1016/S1470-2045(18)30106-2

[35] Robert C, Schachter J, Long GV, et al. Pembrolizumab versus ipilimumab in advanced melanoma. *N Engl J Med*. 2015;372(26):2521–32. 10.1056/NEJMoa1503093. 25891173 10.1056/NEJMoa1503093

[36] Kwak M, Farrow NE, Salama AKS, et al. Updates in adjuvant systemic therapy for melanoma. *J Surg Oncol*. 2019;119(2):222–31. 10.1002/jso.25298. 30481375 10.1002/jso.25298 PMC6330126

[37] Lao CD, Khushalani NI, Angeles C, Petrella TM. Current state of adjuvant therapy for melanoma: less is more, or more is better? *Am Soc Clin Oncol Educ Book*. 2022;42:1–7. 10.1200/EDBK_351153. 35658502 10.1200/EDBK_351153

[38] Balch CM, Gershenwald JE, Soong SJ, et al. Final version of 2009 AJCC melanoma staging and classification. *J Clin Oncol*. 2009;27(36):6199–206. 10.1200/JCO.2009.23.4799. 19917835 10.1200/JCO.2009.23.4799 PMC2793035

[39] Gershenwald JE, Scolyer RA, Hess KR, et al. Melanoma staging: evidence-based changes in the American Joint Committee on Cancer eighth edition cancer staging manual. *CA Cancer J Clin*. 2017;67(6):472–92. 10.3322/caac.21409. 29028110 10.3322/caac.21409 PMC5978683

[40] Bobirca F, Leventer M, Georgescu DE, et al. Variability of sentinel lymph node location in patients with trunk melanoma. *Diagnostics (Basel)*. 2023. 10.3390/diagnostics13172790. 37685328 10.3390/diagnostics13172790 PMC10486776

[41] Federico AC, Chagpar AB, Ross MI, et al. Effect of multiple-nodal basin drainage on cutaneous melanoma. *Arch Surg*. 2008;143(7):632–7. 10.1001/archsurg.143.7.632. (**discussion 637-8**). 18645103 10.1001/archsurg.143.7.632

[42] McHugh JB, Su L, Griffith KA, et al. Significance of multiple lymphatic basin drainage in truncal melanoma patients undergoing sentinel lymph node biopsy. *Ann Surg Oncol*. 2006;13(9):1216–23. 10.1245/s10434-006-9014-z. 16952026 10.1245/s10434-006-9014-z

[43] Uren RF, Howman-Giles R, Thompson JF. Patterns of lymphatic drainage from the skin in patients with melanoma. *J Nucl Med*. 2003;44(4):570–82. 12679402

