# Supplementary material for: Evaluating Variation in Lymph Node Sampling During Sentinel Lymph Node Biopsy for Melanoma
Source: Ann Surg Oncol. 2025 Aug 16;32(13):9660–7. doi: 10.1245/s10434-025-18063-5 (PMC12589253; doi:10.1245/s10434-025-18063-5)

**Supplement:**

**Table 1: Reliability-Adjusted Mean Sentinel Lymph Node Yield by Case Volume for Melanoma of the Trunk and Upper Extremities**

| **Facility Volume Category** | **Sentinel Lymph Node Yield Mean (95% CI)** |
| --- | --- |
| Low (<8) | 2.5 (2.4 - 2.5) |
| Medium (8 - 50) | 2.5 (2.4 - 2.5) |
| High (≥51) | 2.6 (2.5 - 2.7) |

CI, confidence interval

**Figure 1: Reliability-Adjusted Mean Sentinel Lymph Node Yield by Case Volume for Melanoma of the Trunk and Upper Extremities**


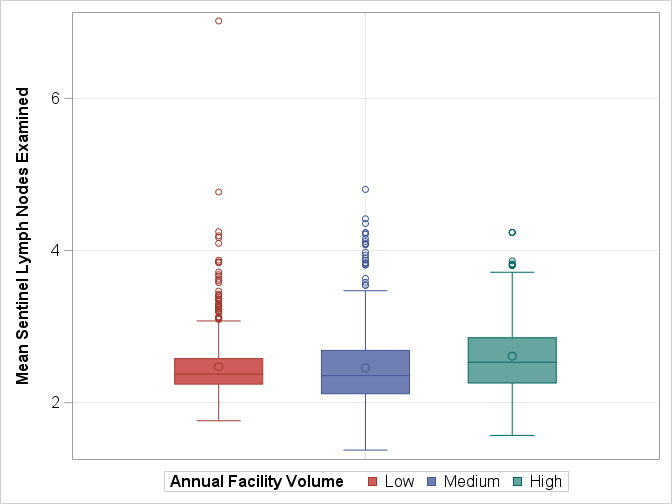


**Table 2: Reliability-Adjusted Nodal Positivity Rates by Facility Type for Melanoma of the Trunk and Upper Extremities**

| **Facility Type** | **Nodal Positivity Rate (95% CI)** |
| --- | --- |
| Unknown | 18.6% (17.9% - 19.2%) |
| Community Cancer | 18.3% (17.9% - 18.6%) |
| Comprehensive Center | 18.2% (17.9% - 18.4%) |
| Academic Center | 19.0% (18.6% - 19.4%) |
| Integrated Network | 18.5% (18.2% - 18.8%) |

CI, confidence interval

**Figure 2: Reliability-Adjusted Nodal Positivity Rates by Facility Type for Melanoma of the Trunk and Upper Extremities**


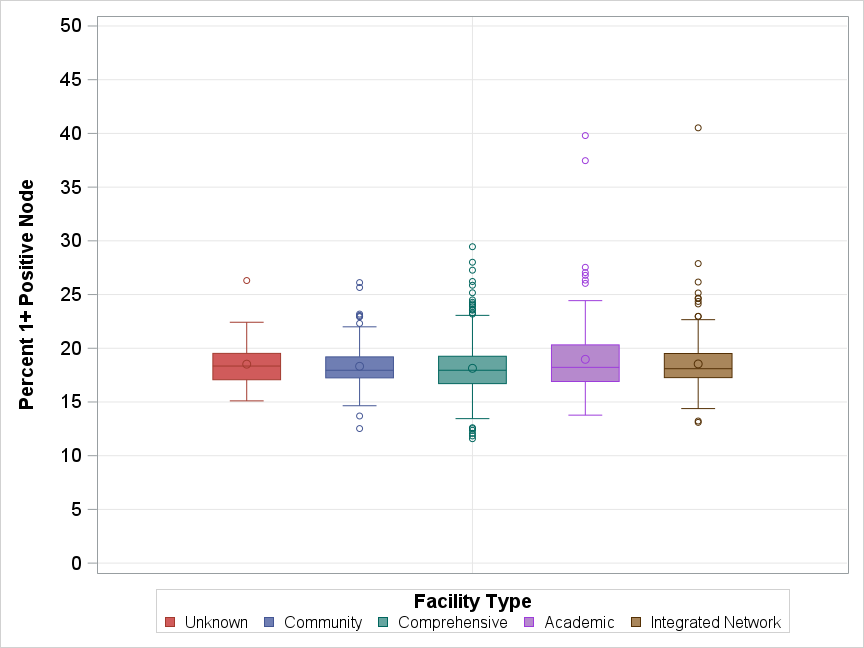

Supplement: Supplementary file 1 — (DOCX 75 kb) [file 10434_2025_18063_MOESM1_ESM.docx]
